# Clinical Evaluation of Fiber-Reinforced Composite Restorations in Posterior Teeth - Results of 2.5 Year Follow-up

**DOI:** 10.2174/1874210601812010476

**Published:** 2018-06-29

**Authors:** Johanna Tanner, Mimmi Tolvanen, Sufyan Garoushi, Eija Säilynoja

**Affiliations:** 1 Department of Prosthetic Dentistry, Institute of Dentistry, University of Turku, Turku, Finland; 2 Department of Community Dentistry, Institute of Dentistry, University of Turku, Turku, Finland; 3 Department of Biomaterials Science, Institute of Dentistry, University of Turku, Turku, Finland; 4 Turku Clinical Biomaterials Center – TCBC, Institute of Dentistry, University of Turku, Turku, Finland; 5 Research, Development and Production Department, Stick Tech LTD – Member of GC Group, Turku, Finland

**Keywords:** Fiber-reinforced composites, Bilayered posterior composite restorations, Clinical study, Polymerization shrinkage, Cavities, Volumetric shrinkage

## Abstract

**Objectives:**

The aim of this study was to evaluate the clinical performance of posterior composite restorations reinforced by bulk base of short-fiber composite (everX Posterior, GC Corporation).

**Methods:**

Thirty-six short fiber-reinforced composite restorations were placed in premolar and molar teeth of 33 patients. Eight of the teeth were vital and 28 were non-vital. Average follow up time of the restorations was 30.6 months (2.5 years), ranging from 16.2 to 51.3 months (1.3 - 4.3 years).

**Results:**

One restoration failed during the follow-up period due to secondary caries, at time point 39.5 months. Three fillings had minor fractures during the follow-up. The overall survival rate of the restorations was 97.2% and success rate (no maintenance needed) was 88.9%, respectively.

**Conclusion:**

Posterior composite restorations with a bulk base of short-fiber composite showed good clinical performance in the short term evaluation.

## 1 INTRODUCTION

Direct composite restorations are commonly used to restore cavities in both anterior and posterior teeth. Clinical performance of composite restorations has improved along with the development of filling material properties. According to the systemic review by Manhart and his colleagues, annual failure rates are in the range of 0-7% [1]. Survival rates of posterior composite restorations vary between 65.2% and 92.5%, with follow-up times ranging from 3 to 17 years [2-7]. The most common reasons for failure of posterior stress-bearing composite restorations are secondary caries, bulk fractures, marginal deficiencies and wear [1, 5, 8]. Direct composite restorations are sensitive to operation technique and one of the factors influencing this is polymerization shrinkage. Volumetric shrinkage of dental composites has been reported to range from 2 to 6% [9]. Shrinkage of the composite causes stress in the adhesive interface between the restoration and the surrounding tooth tissues. This may lead to marginal leakage, postoperative sensitivity and secondary caries. It is recommended to use an incremental layering technique while placing a composite restoration, in order to decrease the polymerization stress, avoid gap formation and to achieve better mechanical properties [10-12]. This technique is however time consuming and may be difficult to perform accurately. To simplify restorative procedures and save chair-side time, so called bulk-fill materials have been developed [13]. These materials are claimed to exhibit lower polymerization shrinkage and improved polymerization kinetics than conservative incremental–technique materials, enabling the placement and curing of the material in layers up to 4 mm [14-17]. Results obtained from laboratory studies have been contradictory, with some materials performing better than others [14, 17-19]. The filling technique and composite has been shown to have a great impact on the adhesion of restorative composites, in particular in high C-factor cavities [18].

Recently, a composite filling material reinforced with short glass fibers (introduced as Xenius base, later named as everX Posterior, GC Corporation) was introduced with an indication as a dentine replacing or base filling material in posterior stress bearing restorations. The material is composed of short, randomly oriented glass fibers in a Bis-GMA, TEGDMA resin matrix. Short Fiber-Reinforced Composite (SFRC) adheres well to cavity walls and the overlaying composite, transferring occlusal loads evenly to the tooth [20, 21]. Light transmission through fibers and resulting increased polymerization depth, allow for a simplified bulk-filling technique. Packing the material into the cavity forces the randomly oriented fibers perpendicular to the axial cavity walls. Its volumetric shrinkage is significantly lower compared to other composite materials [22]. The polymerization contraction of SFRC is reduced in the direction of the long axis of the fibers [23]. Laboratory research has shown that, the use of a bilayered structure consisting of a fiber-reinforced composite substructure combined with a surface layer of conventional restorative composite increases the fracture load of a restoration [19, 24-28]. Composite crowns in endodontically treated molars were also significantly reinforced with SFRC core restoration [29]. In a study comparing mechanical properties of bulk-filling materials, SFRC material was found to have a higher fracture toughness and flexural strength, and showed less shrinkage strain, than other bulk-filling composites tested [26]. In 2012, authors have published one-year results of a preliminary clinical study, where the studied material was found to be clinically applicable and showed good clinical performance in restoring large coronal defects in both vital and non-vital teeth [30]. This pilot study used the same materials and a similar study design. Ten of the patients and restorations in the present follow-up study were involved also in the pilot study, reporting one-year follow-up data. Thus, the aim of this study was to evaluate the clinical performance of posterior composite restorations reinforced by a bulk base of SFRC composite in a practice based study.

## 2 MATERIALS AND METHODS

Thirty-three patients received altogether thirty-six SFRC restorations. The restorations were placed by four experienced clinicians in a private practice-based study design. The study protocol was approved by the Joint Commission on Ethics of the Turku University and the Turku University Central Hospital (20.6.2006). For this study, patients were selected according to pre-determined inclusion criteria among the registers of private practice dental offices in Finland from January 2009 to May 2011. All teeth were in occlusion and had at least one proximal contact with an adjacent tooth. Patients with extremely poor oral hygiene, heavy bruxism habits or periodontal problems were excluded.

The restored teeth were predominantly molars (n=28), seven restorations were placed in premolars and one restoration in a canine. Eight (8) of the teeth were vital and 28 were non-vital (root-canal treated). The extent of restorations ranged from small to large, with 18 of the restorations covering 1-3 surface (small) and 18 covering 4-5 surfaces (large) of the tooth. Thirteen (13) of the patients were female and 20 were male. Study data is described in Table **1**.

### 2.1 Restorative Procedures

Before the start of the study, the operative procedure was thoroughly discussed with the dentists. Three different adhesive systems were used randomly. Single-step self etch primer and bond (Vivapen, IvoclarVivadent, Schaan, Liechtenstein) and two-steps self etch primer and bond (Clearfil SE bond, Kuraray, Tokyo, Japan) and three-steps etching, primer and bond (Scotchbond multipurpose adhesive, 3M ESPE, USA). Bonding agents were used according to manufacturer's instructions. The SFRC (everX Posterior, GC Corporation, Tokyo, Japan) was placed and light-cured according to an incremental technique. Care was taken not to extend the SFRC base filling into the outer margins of the cavities. In all restorations the SFRC bulk base was covered with a surface layer of hybrid composite resin (1-2 mm). The composites used were: Estelite (Tokuyama, Japan), Clearfil Majesty Posterior (Kuraray, Japan), Z250 (3MESPE, USA), Z100 (3MESPE, USA), and Synergy (Coltène/Whaledent, Altstätten, Switzerland). Occlusal adjustment, finishing and polishing procedures were carried out predominantly at same visit.

### 2.2 Evaluation

**Fig. (1):**
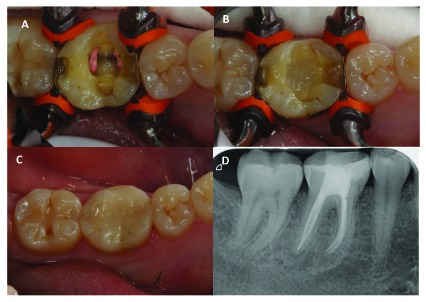
Clinical photographs (**A**, **B** and **C**) and radiograph (**D**) presenting a root-canal treated molar tooth restored with a bilayered restoration using a SFRC bulk base filling and a conventional composite superficially.

Restorations were evaluated at yearly re-call visits of the patients. Patients were instructed to contact the clinicians in case of adverse effects or failures. Evaluation was performed with modified USPHS criteria (Table **2**). Clinicians were calibrated with regard to evaluation criteria through discussions. The survival probability was analyzed at two different levels: success (restoration performing without any failures described above) and survival (restoration performing, but has required some repair). Photograph and X-ray records of restorations were used (Fig. **1**).

### 2.3 Statistical Analysis

Differences in the evaluation criteria (gender, age, tooth type, jaw, endodontic status and number of surfaces restored) according to anatomical form and marginal adaptation at 2.5 year time-point in average were evaluated using cross-tabulation and the Likelihood ratio test. Differences in follow-up time according to anatomical form and marginal adaptation were evaluated with t-test. Separate survival curves for levels “success” and “survival” were estimated using the Kaplan-Meier method. Statistical significance level was set at 0.05. Analysis was conducted using *SPSS* statistics 22.

## 3 RESULTS

Average follow-up time of the restorations was 30.6 months (2.5 years), ranging from 16.2 to 51.3 months (1.3 - 4.3 years). One restoration (2.7%) failed during the follow-up period, due to secondary caries, at time point 39.5 months. The failed restoration was a one-surface filling in a vital mandibular molar tooth of a male patient, in the age cohort 30-40 years. Three fillings had minor fractures during the follow-up. The fractures were superficial chippings of the particulate filler composite and could be treated through finishing and polishing procedures. Fractures occurred in different composite brands. All three fractured fillings continued to function throughout the study. Marginal discoloration was found in seven cases. The discoloration was superficial and could be polished away. The distribution of findings with regard to evaluation criteria is presented in Table **3**. Overall survival rate of the restorations was 97.2% (A and B scores accepted, “survival”). When only A scores were regarded as acceptable (“success”), the calculated overall success rate was found to be 88.9%.

Distribution of patient and restoration variables according to anatomical form and marginal adaptation is reported in Table **3**. Figs. (**2** and **3**) illustrate the cumulative survival function for levels “success” and “survival”.

## 4 DISCUSSION

This case-series study investigated the clinical performance of thirty-six short fiber reinforced posterior composite restorations (bilayered restorations). With an overall survival rate of 97%, restorations were found to perform very well during the relatively short follow-up period. Only one restoration was lost and 3 needed minor adjustments during the follow-up.

The present study is executed as a practice-based prospective case-series study. The main drawbacks of the study design are the lack of a control group and randomization. Some variations in the selection of patients and manufacturing of the restorations may have occurred, despite the fact that all dentists were calibrated with regard to study protocol at the onset of the study. Compared to a randomized controlled clinical trial this practice-based study offers less power, but simulates the every day clinical challenges better than a study performed in strictly controlled circumstances.

The follow-up time in the study is relatively short, on average only 2.5 years. Possible clinical failures will need a longer service-time to occur than this and it is evident that the results presented now can only be regarded as preliminary in the clinical perspective. This clinical technique is however novel and also short-term clinical data can thus be considered to give valuable information. Studies published on the material in question report clinical follow-up data up to 12 months so far [30].

Anatomical form and marginal adaptation parameters were evaluated clinically. Patient age and the extent of the restoration (number of surfaces restored) had a significant effect on anatomical form. The extent of restoration affected also the presence of marginal discoloration. All failures in anatomical forms occurred in the age cohort 30-40 years. The small number of cases, that is 4 cases, however, prohibits us to make any assumption on causality. More marginal discoloration was observed in 4-5 surface restorations compared to small 1-3 surface restorations. Larger restorations are found to have higher annual failure rates than restoration with less surfaces involved [31]. The larger the cavity, the more technique sensitive is the actual restorative procedure. In large cavities, there is more likely also a lack of enamel support at margins. Bond strength to dentin is lower than to enamel and adhesive interfaces are known to deteriorate much quicker when bonded to dentin than to enamel [32, 33]. Restorations in the present study showed, however, better marginal adaptation than the results of Palaniappan *et al*., who investigated clinical performance and wear of hybrid composite restorations in a short time follow-up study [34]. Their data showed B scores in most of the followed restorations in the three-year follow-up investigation, whereas a majority of cases in the present study received an A score. A positive influence on marginal adaptation has been shown also *in vitro*. Schwendicke and co-workers investigated margin integrity and mechanical properties of large posterior composite restorations *in vitro* and found that using SFRC base-filling resulted in good margin quality and increased fracture resistance of molar teeth compared to particulate filler composite (Z250) alone [27].

Research conducted on posterior composite restorations, reveals fractures, marginal discoloration and changes in surface appearance of the restorations even after short-term clinical use [1, 35]. Polymerization shrinkage may cause marginal leakage in the interface of tooth and restoration, which can result in secondary caries. Due to internal constraint factor of fiber composite, the polymerization shrinkage can be lowered by using fibers as fillers in the composite [22, 23]. In the present study, we observed very little marginal discoloration and no fractures leading to renewal of restorations. Our clinical findings are in line with the *in vitro* data reporting on a diminished polymerization shrinkage and better fracture toughness of SFRC compared to conventional particulate filled composites [26].

Majority of the teeth restored and followed in the present study were non-vital molars. With curing depth up to 4 millimeters, SFRC offers an efficient solution to restore the pulpal cavity in a root canal treated tooth. Molars, being larger in volume than premolars and incisors, more often present with a sufficient amount of remaining coronal tooth structure for the application of a direct adhesive technique. The lower polymerization shrinkage of SFRC compared to particulate filler composite, causes less shrinkage stress in the interface between the filling and tooth tissue. A lower risk for cuspal deflection and dentin fractures may thus be advocated. In the present study, no difference was found in the survival or performance of restorations in vital *versus* non-vital teeth. Previous studies have found a lower survival percentage and more technical failures in the restorations made in non-vital teeth compared to vital ones [36]. The small number of vital teeth in the present study may in part explain our lack to demonstrate any differences. In an *in vitro* experiment, SFRC bulk base-filling was found to significantly reinforce a composite crown in a root canal treated molar [29]. Multi-directional short fibers had a better reinforcing effect than unidirectional continuous fibers. Fracture propagation and orientation were also found to be more favourable in the presence of SFRC filling. More recently, also Ozsevlic *et al.,* and Yasa *et al.,* compared SFRC and conventional composite in restoring endodontically treated molars. They both found highest fracture loads for teeth restored with SFRC in comparison with conventional composite alone [37, 38]. These *in vitro* findings are corroborated by the clinical observations in the present study. Theoretically, the function of the SFRC bulk base is assumed to be based on supporting the superficial particulate filled composite and behaving as a crack arrest barrier. In other words, this bilayered restoration is able to mimic the natural behaviour of enamel and dentin. To the author's knowledge, this SFRC might be the only available composite resin that is capable of bio-emulation by structurally mimicking dentin in its behaviour under load.

We conclude that direct composite restorations reinforced with SFRC-base (bilayered technique) show a good clinical performance in the short term evaluation. This technique is clinically applicable and might offer a cost-effective way to restore large posterior cavities in vital and non-vital teeth.

## Figures and Tables

**Fig. (2):**
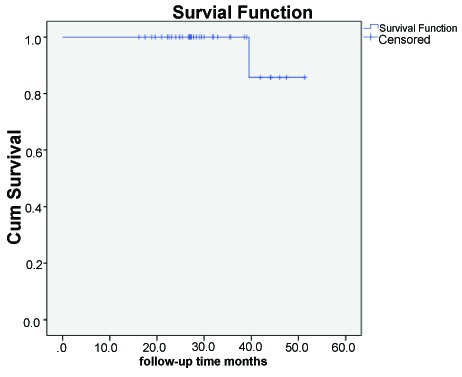
Cumulative survival function for the level “Survival” (restoration performing, but has required some repair). Average follow up time of the restorations was 2.5 years, ranging from 1.3 - 4.3 years.

**Fig. (3):**
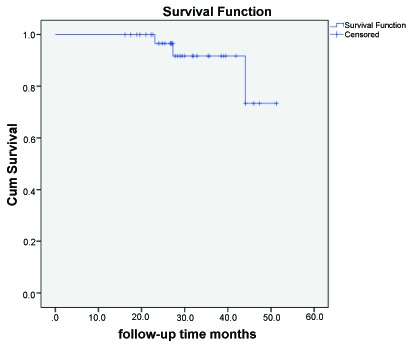
Cumulative survival function for the level “Success” (restoration performing without any failures). Average follow up time of the restorations was 2.5 years, ranging from 1.3 - 4.3 years.

**Table 1:** Characteristics of patients and restorations.

–	N	Vital (N)	Non-vital (N)
Patients	33	–	–
Female	13	–	–
Male	20	–	–
Restorations	36	8	28
Molars	28	7	24
Premolars	7	1	4
Canine	1	1	–
Maxilla	12	–	–
Mandible	24	–	–
1-3 Surface restorations	18	5	13
4-5 Surface restorations	18	0	18

**Table 2:** Modified USPHS criteria for the clinical evaluations of restorations.

Category	Score		Criteria
–	Acceptable	Unacceptable	–
–	–	–	–
Anatomical form	A	–	Restoration's contour is continuous with tooth anatomy
–	B	–	Restoration is slightly over or under contoured; minor chipping or occlusal height slightly reduced
–	–	C	Restoration is undercontoured; dentin or base exposed; contact is faulty
–	–	–	–
Marginal adaptation	A	–	Excellent continuity at resin-enamel interface; no ledge formation; no discoloration
–	B	–	Slight discoloration at resin-enamel interface; ledge at interface
–	–	C	Moderate discoloration at resin-enamel interface, cannot be polished away; obvious crevice at margin
–	–	D	Recurrent decay at margin
–	–	–	–
Secondary caries	A	–	No visible caries
–	–	B	Caries contiguous with the margins of the restoration
–	–	–	–

**A**: alpha, **B**: bravo, **C**: charlie, **D**: delta

**Table 3:** Distribution (%) of patient and restoration variables according to anatomical form and marginal adaptation.

–		Anatomical Form			Marginal Adaptation		
		A	B	*p*	A	B	*p*
		n=32	n=4		n=29	n=7	
–	–	%	%	–	%	%	–
Gender	Female	47	25	0.394	41	57	0.456
–	Male	53	75	–	59	43	–
Age	20–30	9	0	<0.001	10	0	0.197
	30–40	3	100		14	14	
	40–50	19	0		21	0	
	50+	69	0		55	86	
Tooth type	molar	75	100	0.341	80	71	0.665
	Premolar	22	0		17	29	
	Canine	3	0		3	0	
Jaw	Mandible	66	75	0.702	69	57	0.557
–	Maxilla	34	25	–	31	43	–
Endodontic	Non-vital	78	75	0.889	79	71	0.66
Status	Vital	22	25	–	21	29	–
Surfaces restored	1	3	25	0.019	3	14	0.188
	2	9	0		10	0	
–	3	41	0		41	14	
–	4	16	75		21	14	
–	5	31	0		21	57	
Mean follow-up time		30.2	33.5	0.501	29.7	34.3	0.23
